# Two-stitch versus one-stitch cervical cerclage in women with high risk for preterm birth: a stratified exploratory randomized controlled trial in China

**DOI:** 10.1186/s12884-026-08809-8

**Published:** 2026-02-16

**Authors:** Zhi-Min Xu, Yi-Jing Zheng, Wen-Xin Yan, Cai-Hong Jiang, Hai-Bo Li, Jun Zhang, Mian Pan

**Affiliations:** 1 Department of Obstetrics and Gynecology, Fujian Maternity and Child Health Hospital, College of Clinical Medicine for Obstetrics & Gynecology and Pediatrics, Fujian Medical University, Fuzhou, China; 2 Department of Medical Ultrasonics, Fujian Maternity and Child Health Hospital, College of Clinical Medicine for Obstetrics & Gynecology and Pediatrics, Fujian Medical University, Fuzhou, China; 3 Division of Birth Cohort Study, Fujian Maternity and Child Health Hospital, College of Clinical Medicine for Obstetrics & Gynecology and Pediatrics, Fujian Medical University, Fuzhou, China

**Keywords:** Cervical insufficiency, Sonographic short cervix, Cervical cerclage, Two-stitch, Randomized controlled trial, Preterm birth

## Abstract

**Background:**

Cervical insufficiency or sonographic short cervix is a major cause of preterm birth (PTB). The efficacy of two-stitch versus one-stitch cerclage remains controversial, with limited evidence from randomized controlled trial (RCT), especially stratified by indication.

**Methods:**

We conducted a single-centre, stratified, exploratory RCT at Fujian Maternal and Child Health Hospital, Fuzhou, China. Women with singleton pregnancies were enrolled into two parallel cohorts: a therapeutic cohort (ultrasound-indicated; cervical length ≤ 25 mm at 16–28 weeks) and an emergency cohort (physical examination-indicated; painless cervical dilatation with or without membrane exposure at 16–28 weeks). Within each cohort, participants were randomly assigned (1:1) to receive McDonald cerclage with either two-stitch or one-stitch. The primary outcome was spontaneous PTB < 34 weeks. RR with 95% CI was calculated as the primary measure of effect size between groups.

**Results:**

Between June 2022 and December 2024, 100 women were enrolled and stratified (therapeutic, *n* = 50; emergency, *n* = 50). In the intention-to-treat (ITT) analysis, there was no significant difference in the primary outcome of spontaneous PTB < 34 weeks between the two-stitch and one-stitch groups in the therapeutic cohort (16.0% vs 0.0%; RR not calculable; *p* = 0.110) or the emergency cohort (40.0% vs 48.0%; RR 0.83, 95% CI 0.44–1.57; *p* = 0.569). However, an exploratory analysis of the emergency cohort revealed that the two-stitch technique was associated with a reduced incidence of PTB < 28 weeks (12.0% vs 40.0%; RR 0.30, 95% CI 0.09–0.96; *p* = 0.024). Neonatal survival rates did not differ significantly in either cohort.

**Conclusions:**

In the therapeutic setting, the two-stitch technique was not superior to the one-stitch approach. For emergency cerclage, while the primary outcome was not significantly reduced, a secondary analysis showed an association between the two-stitch technique and a reduced incidence of extreme PTB < 28 weeks. This hypothesis-generating finding suggests a potential benefit in preventing extreme PTB < 28 weeks and must be validated in larger, definitive trials.

**Trial registration:**

The Chinese Clinical Trial Registry (ChiCTR), Identification Number ChiCTR2200058540. https://www.chictr.org.cn/bin/project/edit?pid=159942.

**Supplementary Information:**

The online version contains supplementary material available at 10.1186/s12884-026-08809-8.

## Background

Cervical cerclage is the surgical treatment for women with cervical insufficiency or sonographic short cervix [1–4]. However, not all women who undergo cervical cerclage have a successful outcome. Two-stitch cervical cerclage is a potential treatment for cervical insufficiency and sonographic short cervix. Some operators opt for a double cerclage by sewing an additional suture above the first one to strengthen the cervical resistance [5]. Some operators re-suture below the first one to reshape the external cervical os and facilitate the formation of a cervical mucus plug to lower the risk of retrograde infection of the amniotic cavity [6, 7].

Despite the proposed mechanical and biological rationales for the two-stitch technique, its clinical efficacy remains controversial. The majority of retrospective evidence, including studies that enrolled women based on both history and ultrasound indications, found no significant improvement in pregnancy outcomes when comparing two-stitch to one-stitch cerclage [8, 9]. Indeed, one of these studies suggested that any potential difference in preterm birth (PTB) incidence < 28 weeks warranted further investigation in a larger cohort [8]. A subsequent randomized controlled trial (RCT) reinforced these findings, demonstrating that placing a second suture to retain the mucus plug did not prolong gestational age (GA) at delivery, reduce neonatal intensive care unit (NICU) days, or improve neonatal mortality [6]. Another retrospective study that analyzed women collectively (based on history, ultrasound, or physical exam indications) also found no difference in the effect of a single or double cerclage on preventing PTB [5]. In contrast, a retrospective study focused on emergency cerclage and a single-center prospective observational study of ultrasound-indicated cerclage reported superior outcomes for the two-stitch technique in their respective populations [7, 10]. The apparent contradiction in these findings may be attributed to heterogeneity in patient populations and cerclage indications. A systematic review offered a potential resolution, proposing that a second suture should be considered when the initial placement is too low or the cerclage position is less than 2 cm from the external cervical os [11].

In summary, there is currently a lack of stratified randomized controlled trials to evaluate the efficacy of two-stitch versus one-stitch cerclage specifically for therapeutic cervical cerclage (ultrasound-indicated) and emergency cervical cerclage (physical examination–indicated). We hypothesized that the two-stitch technique would be superior to the one-stitch technique in reducing spontaneous PTB < 34 weeks, with a greater effect size anticipated in the emergency cerclage cohort.

## Methods

### Study design and setting

We conducted a ‌single-center, stratified (therapeutic and emergency trial), exploratory RCT at Fujian Maternal and Child Health Hospital in Fuzhou, Fujian Province, China, a tertiary referral center with 1,000 beds and 20,000 annual deliveries. The trial was registered prospectively on the Chinese Clinical Trial Registry (ChiCTR2200058540; April 10, 2022) and approved by the Ethics Committee of Fujian Maternity and Child Health Hospital (Affiliated Hospital of Fujian Medical University) (No. 2022YJ012; March 22, 2022). The trial was monitored by the Fujian Maternal and Child Health Hospital Ethics Committee, which reviewed annual progress reports and any reported serious adverse events. It was conducted in accordance with the 1964 Helsinki declaration and its later amendments or comparable ethical standards. Written informed consent was obtained from all participants. This study was conducted and reported in accordance with the CONSORT guidelines.

### Participants‌

#### Inclusion criteria‌

Women were eligible if they met ‌either category‌:

1. Therapeutic cohort (Ultrasound-indicated cerclage‌): Singleton pregnancy with:
  1. History of spontaneous PTB < 34 weeks ‌and cervical length ≤ 25 mm at 16–28 weeks;
  2. No prior PTB but CL ≤ 25 mm ‌‌and ‌progressive shortening at 16–28 weeks.

2. Emergency cohort (physical examination-indicated cerclage): Singleton pregnancy with painless external os dilation with or without the membranes being exposed to the vagina at 16–28 weeks.

#### Exclusion criteria‌

Transabdominal cerclage, using a pessary for cervical shortening, fetal anomalies, medically indicated preterm delivery, lost to follow-up or incomplete documentation of delivery outcomes.

### Randomization and blinding

#### Stratification and Randomization

Participants were primarily stratified by clinical indication (Therapeutic cerclage cohort vs. Emergency cerclage cohort, n=50 per stratum). Within each stratum, they were randomly assigned (1:1) to receive either the one-stitch or two-stitch technique (n=25 per group).

The allocation sequence was computer-generated by an independent statistician using the SPSS 26.0 (IBM SPSS, Inc, Armonk, NY) with permuted blocks of sizes 4 and 6.

#### Concealment mechanism

For each eligible participant, this independent research nurse provided the corresponding sequentially numbered, opaque, sealed envelope directly in the operating room. The envelope was opened by the surgeon at the specified time (after anesthesia preparation and final decision to operate). The surgeon did not have access to the master set of envelopes, and only one envelope was in the operating room at any given time.

Stratum-specific randomization lists were maintained independently to prevent cross-stratum contamination.

#### Blinding

The researcher who obtained the randomization information did not participate in any stage of the trial.

##### Participants and nursing staff‌

Masked to group assignment. Blinding of participants and nursing staff was achieved by using identical postoperative care protocols, and by not documenting the number of sutures in the patient's accessible medical notes.

##### Surgeons‌

Unblinded due to procedural requirements but mandated to follow standardized operative checklists and excluded from outcome evaluations.

##### Outcome assessors‌

Outcome assessors were blinded to the treatment allocation. The primary outcome was determined based on objective criteria, which are not subject to interpretation based on the surgical technique.

This design ensures that potential confounding from clinical indication type was controlled through stratification, while preserving random allocation properties within each prognostic subgroup [12].

### Intervention

1. Preoperative preparation: The basic condition of the patient was fully assessed before surgery, and preoperative examinations were completed to exclude surgical contraindications. The patient was informed of the condition and the risks related to the surgery, and a surgical informed consent form was signed.
2. Surgical procedure: All patients underwent the McDonald cervical cerclage procedure performed by the same experienced chief obstetrician (M.P., with over 10 years of experience in the technique), using a 5-mm wide Mersilene tape (Johnson & Johnson Ethicon, RS22). The cervix was exposed using a vaginal retractor.

#### Reduction of Bulging Membranes

If the amniotic sac protruded beyond the external cervical os, it was gently reduced by placing the patient in a steep Trendelenburg position and applying steady, gentle pressure with a moistened gauze on a sponge stick. The reduction was continued until the membranes were repositioned to the level of the internal cervical os. Following the circumferential placement of the cerclage suture, the knot was securely tied while simultaneously withdrawing the gauze that had been used for membrane support.

#### Suture Placement of One-stitch Group

The Mersilene tape was passed through the cervical stroma at the level of the cervicovaginal junction (the bladder reflection). The needle was passed from anterior to posterior, aiming to incorporate a substantial bite of cervical stroma while avoiding mucosal penetration into the endocervical canal. The suture was placed circumferentially in a purse-string manner, with bites spaced approximately 1–1.5 cm apart. The knot was tied firmly, but without causing tissue strangulation, anteriorly, leaving a suture loop that admitted the tip of a fine hemostat. 

#### Suture Placement of Two-stitch Group

**Fig. 1:**
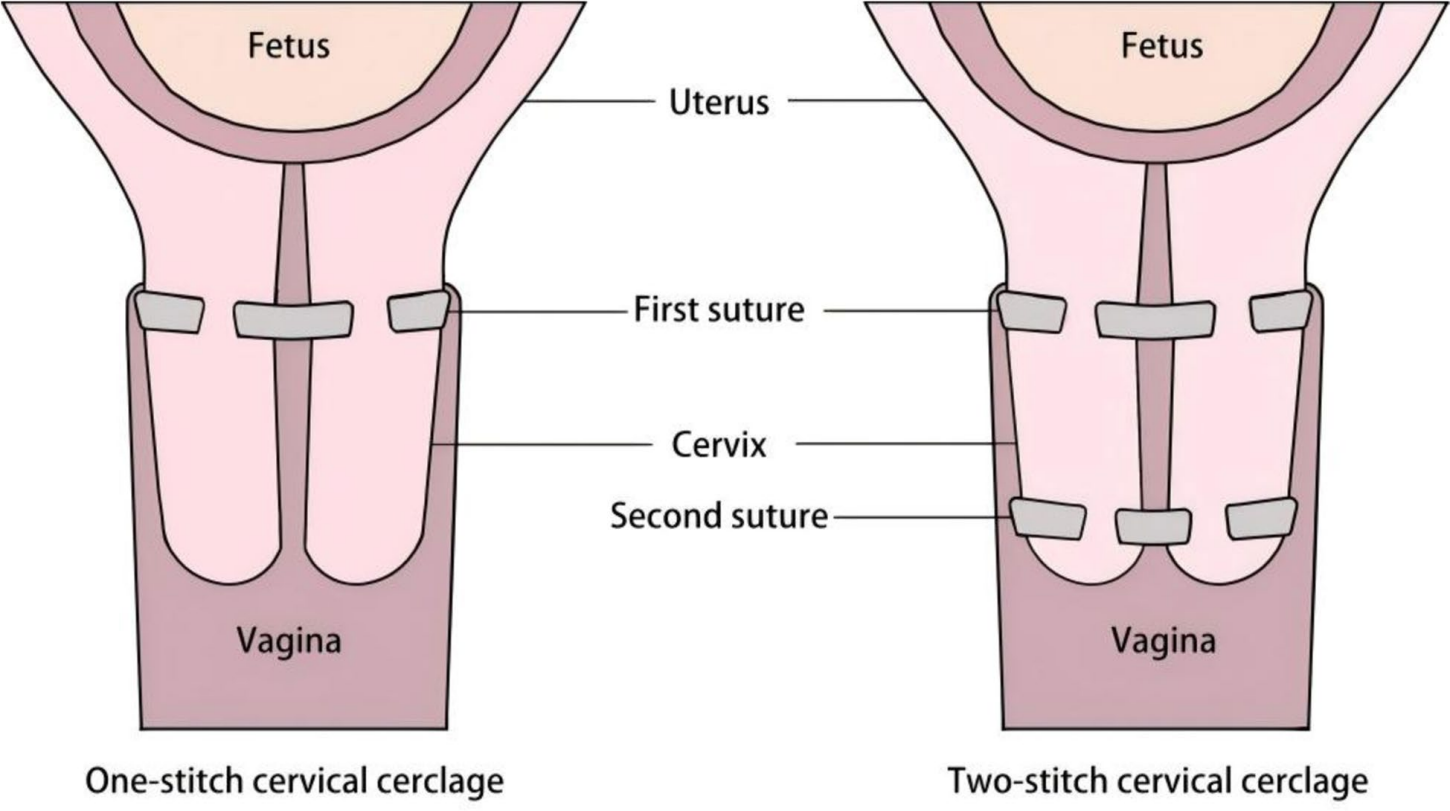
Schematic diagram of the one-stitch and two-stitch cervical cerclage techniques

The first cerclage suture (S1) was placed in an identical manner to the one-stitch group, at the highest possible level close to the internal os. The second suture (S2) was then placed circumferentially below the first, near the external cervical os. The distance between S1 and S2 was approximately 1–1.5 cm. The S2 suture was placed using the same technique regarding depth and spacing, with the specific aim of actively closing the external os to facilitate the reformation of a mucus plug. Both knots were tied anteriorly (Fig. 1).

3. Postoperative management: Patients were placed in a Trendelenburg position for bed rest after surgery. Perioperative antibiotics were used to prevent infection, and external genital hygiene was maintained. Infection indicators, uterine contractions, vaginal discharge, and subjective symptoms of the patient were closely monitored. The changes in the cervix were dynamically monitored by ultrasound. Appropriate tocolytic agents were used according to GA and uterine contraction symptoms. According to our institutional protocol at the time of the study, which was consistent with the then-current ACOG guidelines [13], a single course of antenatal corticosteroids was administered to women between 24 and 33^+6^ weeks of gestation who were considered at high risk of delivery within the next 7 days. The regimen consisted of dexamethasone, administered as four 6-mg intramuscular doses given 12 h apart. This assessment was based on clinical judgment of preterm labor. The cervical cerclage was usually removed at 36–37 weeks of gestation. In case of definite signs of infection, preterm premature rupture of membranes (PPROM), inevitable miscarriage or preterm labor unresponsive to tocolytic agents, the cerclage should be urgently removed in the emergency department. The timing of cerclage removal should be grasped to reduce the occurrence of cervical laceration.

## Outcome measures

### Primary outcome

#### Spontaneous PTB < 34 weeks‌

##### Definition

Delivery occurring before 34 completed weeks of gestation due to spontaneous PTB or PPROM, excluding iatrogenic PTB.

##### GA determination

Gestational dating was standardized using first-trimester crown-rump length (CRL) measurements (11–13 weeks). When discrepancy > 7 days existed between last menstrual period and ultrasound dating, CRL-based dating was prioritized.

### Secondary outcomes

Secondary outcomes included: GA at delivery; PTB < 28 weeks, PTB < 32 weeks, PTB < 37 weeks; gestational latency‌(cerclage-to-delivery interval); post-cerclage cervical closure length (CCL); positive rate of cervical secretion culture after cerclage; chorioamnionitis (placental pathology using Amsterdam criteria). Outcomes for surviving neonates included: neonatal birth weight, apgar score, NICU admission rate and NICU admission days, and neonatal complications (respiratory distress syndrome, intraventricular hemorrhage, necrotizing enterocolitis, sepsis, retinopathy of prematurity, and bronchopulmonary dysplasia).

Adverse events were monitored and recorded from randomization through postpartum hospital discharge. Specifically, premature rupture of membranes (PROM) and PPROM were events of special interest. All adverse events were systematically assessed at each postoperative follow-up visit and at delivery.

### Statistical analysis

The sample size calculation was based on the primary outcome measure. Data from previous cohort studies and patients who underwent transvaginal cervical cerclage at the trial center were taken into account. The expected incidence of PTB < 34 weeks of gestation was 42.1% for the two-stitch cerclage group and 89.5% for the one-stitch cerclage group [10]. Assuming a significance level of 0.05 and a power of 90%, a sample size of 44 was required. Considering an anticipated dropout rate of 5%, a minimum of 46 patients were needed to be enrolled in the study. We acknowledge that this calculation was based on an optimistic effect size, and this pilot trial is therefore likely underpowered to detect the smaller, clinically relevant differences that are more plausible.

Continuous variables were assessed for normality using the Shapiro–Wilk test. Normally distributed data are presented as mean ± standard deviation (SD) and analyzed with independent samples t-tests to estimate mean differences with 95% confidence interval (CI). Non-normally distributed data are expressed as median (Q1, Q3) and compared using Wilcoxon rank-sum tests, with median differences and 95% CIs calculated via the Hodges-Lehmann method. Categorical variables were compared using χ^2^ tests (when all expected frequencies ≥ 5) or Fisher's exact tests (when any expected frequency < 5). For dichotomous outcomes, relative risk (RR) with 95% CI was calculated as the primary measure of effect size between groups.

The primary analysis was performed according to the ITT principle, including all randomly assigned participants. For the two participants in the therapeutic cohort with missing data on the primary outcome, a conservative approach was applied by imputing them as having experienced the event (spontaneous PTB < 34 weeks). A supporting per-protocol (PP) analysis, which excluded these two participants, was also conducted. Given the exploratory nature of this pilot trial and the multiple secondary outcomes reported, no adjustments for multiple comparisons were made, and p-values for secondary outcomes should be interpreted with caution.

No subgroup or sensitivity analyses were pre-specified in the statistical analysis plan for this initial trial. All analyses presented are therefore considered primary or exploratory. No interim analyses for efficacy or futility were planned or conducted. A two-sided p-value of less than 0.05 was considered statistically significant. All statistical analyses were performed using SPSS 26.0 (IBM SPSS, Inc, Armonk, NY).

### Protocol availability

The trial protocol and detailed statistical analysis plan were approved by the institutional ethics committee and are publicly available on the Chinese Clinical Trial Registry (ChiCTR) website [https://www.chictr.org.cn/bin/project/edit?pid=159942], identifying number ChiCTR2200058540. The full study protocol is provided as Additional File 1 with this manuscript. No important changes were made to the trial methods or outcomes after the trial commenced. The analysis presented here is in accordance with the pre-specified statistical analysis plan.

## Results

**Fig. 2:**
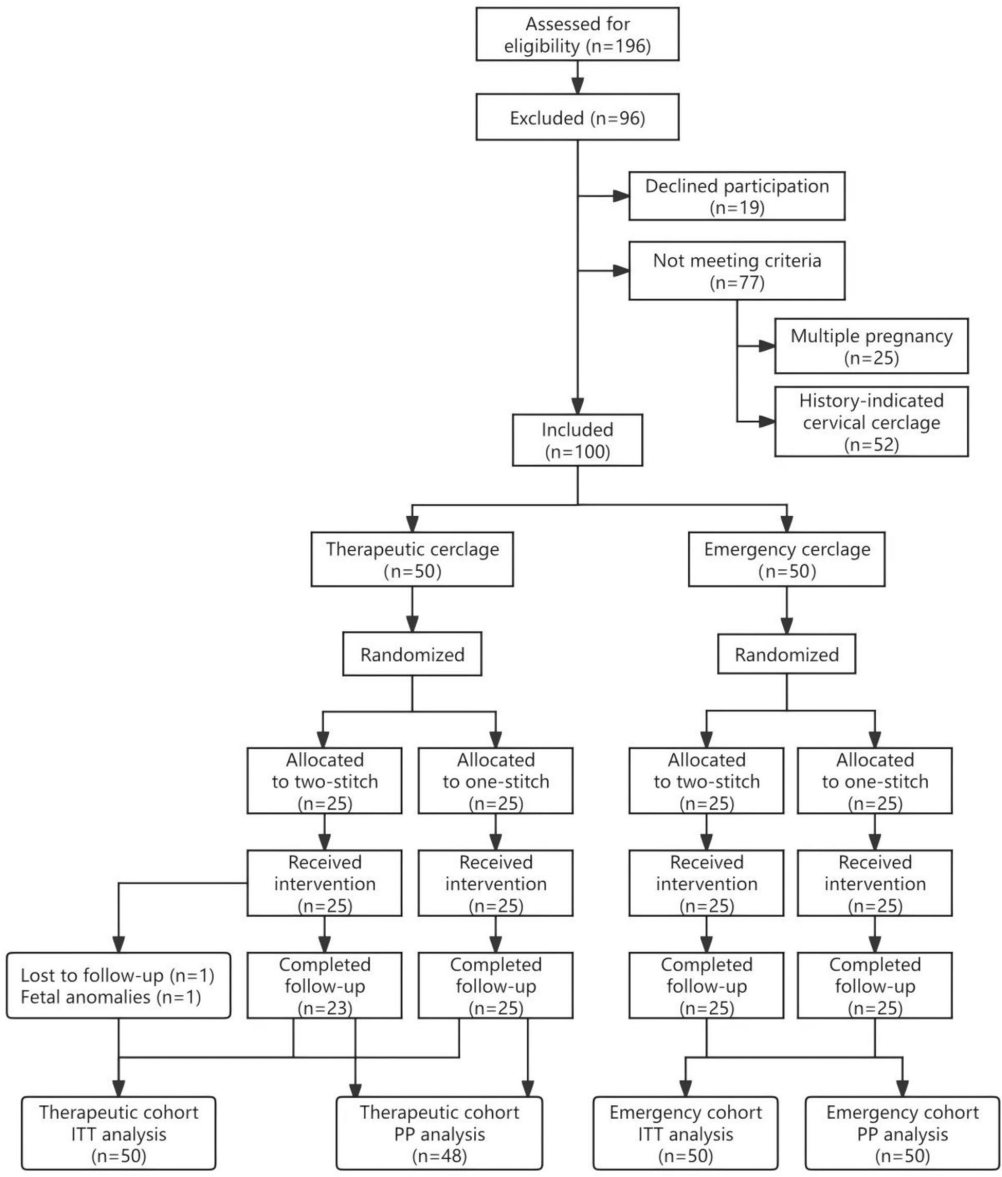
Participant flow diagram. Abbreviations: ITT, intention-to-treat; PP, per-protocol

The flow of participants through the trial is summarized in Fig. 2. The trial concluded as planned upon reaching the pre-determined sample size. Briefly, 100 women were enrolled, stratified, and randomized, constituting the ITT population. All procedures were performed successfully as allocated in the 100 participants who received surgery. For the primary outcome, we conservatively imputed the two excluded participants in the two-stitch group of the therapeutic cohort as having experienced the event. The PP analysis, which excluded these two participants, included 48 women in the therapeutic cohort (one-stitch: *n* = 25; two-stitch: *n* = 23) and all 50 in the emergency cohort.

In the ITT population, maternal demographics and obstetric history were well-balanced between the two-stitch and one-stitch groups in both emergency and therapeutic cerclage cohorts (Table 1). In the emergency cerclage cohort, the two-stitch and one-stitch groups showed comparable maternal age (30.84 ± 4.25 years vs. 32.16 ± 5.06 years; *p* = 0.323). Preoperative cervical dilation was similar between groups (2.00 [1.00, 3.00] cm in both groups; *p* = 0.968), as was the incidence of bulging membranes (72.0% vs. 84.0%; *p* = 0.306). The rates of prior second-trimester loss (28.0% vs. 24.0%; *p* = 0.747) and PTB (0.0% vs. 4.0%; p > 0.999) were similar. Conception via artificial assisted was only reported in the one-stitch group (0.0% vs. 8.0%; *p* = 0.490). Similarly, in the therapeutic cerclage cohort, maternal age was comparable between groups (30.56 ± 4.37 years vs. 30.48 ± 4.54 years; *p* = 0.950), as was Pre-cerclage CCL (1.38 ± 0.63 cm vs. 1.39 ± 0.54 cm; *p* = 0.962). The rates of prior second-trimester loss (48.0% vs. 40.0%; *p* = 0.569) and PTB (20.0% vs. 32.0%; *p* = 0.333) showed no significant differences. Conception via artificial assisted was also similar between groups (4.0% vs. 8.0%; p > 0.999). Concomitant care, including the use of tocolytics and antenatal corticosteroids (Table 1), was administered based on standard clinical protocols. Where data were available, the usage rates did not differ significantly between the two groups within each cohort.

**Table 1:** Characteristics of the study population by suture technique^a^

	**One-stitch**	**Two-stitch**	***P* value**
**Emergency cerclage**	**(*n* = 25)**	**(*n* = 25)**	
Age, year	32.16 ± 5.06	30.84 ± 4.25	0.323
Pre-pregnancy BMI, kg/m^2^	22.96 ± 4.81	24.02 ± 3.26	0.366
Gravity	2.00(2.00, 4.00)	2.00(2.00, 3.00)	0.469
Parity	1.00(0.00, 1.00)	0.00(0.00, 1.00)	0.106
Method of conception			0.490
Natural conception	23(92.0%)	25(100.0%)	
Artificial assisted conception	2(8.0%)	0(0.0%)	
Prior second-trimester loss	6(24.0%)	7(28.0%)	0.747
Prior PTB	1(4.0%)	0(0.0%)	> 0.999
Prior cervical conization surgery	0(0.0%)	0(0.0%)	> 0.999
Cerclage in previous pregnancies	0(0.0%)	0(0.0%)	> 0.999
Preoperative white blood cell,10^9^/L	9.61 ± 1.95	10.76 ± 2.88	0.104
Preoperative CRP, mg/L	4.18(1.17, 8.99)	6.01(2.24, 9.74)	0.869
GA at cerclage, week	23.46 ± 2.97	23.12 ± 2.22	0.647
Cervical dilation, cm	2.00(1.00, 3.00)	2.00(1.00, 3.00)	0.968
Bulging membranes	21(84.0%)	18(72.0%)	0.306
Tocolysis	22(88.0%)	25(100.0%)	0.235
Antenatal corticosteroids	7(28.0%)	6(24.0%)	0.747
**Therapeutic cerclage**	**(*n* = 25)**	**(*n* = 25)**	
Age, year	30.48 ± 4.54	30.56 ± 4.37	0.950
Pre-pregnancy BMI, kg/m^2^	22.66(20.95, 28.85)	22.23(19.96, 25.18)	0.064
Gravity	2(1, 3)	2(1, 2)	0.692
Parity	0(0, 1)	0(0, 1)	0.299
Method of conception			> 0.999
Natural conception	23(92.0%)	24(96.0%)	
Artificial assisted conception	2(8.0%)	1(4.0%)	
Prior second-trimester loss	10(40.0%)	12(48.0%)	0.569
Prior PTB	8(32.0%)	5(20.0%)	0.333
Prior cervical conization surgery	1(4.0%)	0(0.0%)	> 0.999
Cerclage in previous pregnancies	1(4.0%)	1(4.0%)	> 0.999
Preoperative white blood cell,10^9^/L	10.01 ± 2.33	10.99 ± 2.82	0.187
Preoperative CRP, mg/L	4.23(1.96, 6.24)	2.67(0.92, 5.83)	0.303
GA at cerclage, week	24.13 ± 2.39	23.83 ± 2.89	0.687
Pre-cerclage CCL, cm	1.39 ± 0.54	1.38 ± 0.63	0.962
Tocolysis	22(88.0%)	19(76.0%)	0.463
Antenatal corticosteroids	4/25(16.0%)	2/23(8.7%)^b^	0.668

*BMI* body mass index (calculated as weight in kilograms divided by the square of height in meters), *PTB* preterm birth, *CRP* C-reactive protein; GA, gestational age, *CCL* cervical closure length

^a^Values are given as Mean ± SD, Median (Q1, Q3), or as number (percentage)

^b^Denominators for "Antenatal corticosteroids" differ due to missing data on medication administration for 2 participants in the two-stitch therapeutic group. The percentages are calculated based on the available data

In the ITT analysis of the primary outcome, which included all 100 randomized women, there was no statistically significant difference in the incidence of spontaneous PTB < 34 weeks between the two-stitch and one-stitch groups in the therapeutic cohort (16.0% vs 0.0%; RR not calculable; *p* = 0.110) or the emergency cohort (40.0% vs 48.0%; RR 0.83, 95% CI 0.44–1.57; *p* = 0.569). The PP analysis yielded consistent results (Supplementary Table 1).

In the PP analysis of the emergency cerclage cohort, Post-cerclage CCL did not differ significantly between two-stitch and one-stitch groups (2.61 ± 0.83 cm vs 2.45 ± 1.25 cm; mean difference 0.16, 95% CI −0.44, 0.77; *P* = 0.588). However, a significantly higher proportion of patients in the two-stitch group achieved a Post-cerclage CCL ≥ 2 cm (84.0% vs 56.0%; RR 1.50, 95% CI 1.02–2.21; *P* = 0.031). In an exploratory analysis of the PP population, the two-stitch approach was associated with a 70% reduction in delivery before 28 weeks (12.0% vs 40.0%; RR 0.30, 95% CI 0.09–0.96; *P* = 0.024). Nevertheless, no significant differences were observed in terms of GA at delivery, gestational latency, or PTB rates at < 32 weeks, < 34 weeks, and < 37 weeks. For the PP population in the therapeutic cerclage trial, post-cerclage CCL (2.89 ± 0.52 cm vs 3.03 ± 0.61 cm; mean difference −0.14, 95% CI −0.47, 0.19; *P* = 0.409) and the rate of post-cerclage CCL ≥ 2 cm (95.7% vs 96.0%; RR 1.00, 95% CI 0.89–1.12; P > 0.999) were comparable between two-stitch and one-stitch groups. Similarly, there were no significant differences between the two-stitch and one-stitch groups regarding GA at delivery, gestational latency, or PTB rates at < 28 weeks, < 32 weeks, < 34 weeks, and < 37 weeks (Table 2).

**Table 2:** Pregnancy outcomes by suture technique^a^

Variable	One-stitch	Two-stitch	P value	RR or median difference or mean difference (95% CI)
**Emergency cerclage**	**(*n* = 25)**	**(*n* = 25)**		
Gestational latency, week	10.28(2.72, 13.65)	11.86(5.07, 14.72)	0.269	−1.57(−4.57, 1.43)
GA at delivery, week	32.24 ± 6.09	33.49 ± 4.95	0.429	1.25(−1.90,4.41)
GA at delivery < 28 weeks	10(40.0%)	3(12.0%)	0.024	0.30(0.09, 0.96)
GA at delivery < 32 weeks	10(40.0%)	9(36.0%)	0.771	0.90(0.44, 1.83)
GA at delivery < 34 weeks	12(48.0%)	10(40.0%)	0.569	0.83(0.44, 1.57)
GA at delivery < 37 weeks	17(68.0%)	17(68.0%)	> 0.999	1.00(0.68, 1.46)
PPROM	7(28.0%)	10(40.0%)	0.370	1.43(0.65, 3.15)
PROM	8(32.0%)	11(44.0%)	0.382	1.38(0.67, 2.83)
Positive rate of cervical secretion culture after cerclage	8(32.0%)	9(36.0%)	0.765	1.13(0.52, 2.44)
Post-cerclage CCL, cm	2.45 ± 1.25	2.61 ± 0.83	0.588	0.16(−0.44, 0.77)
Post-cerclage CCL ≥ 2 cm	14(56.0%)	21(84.0%)	0.031	1.50(1.02, 2.21)
Chorioamnionitis	13(52.0%)	10(40.0%)	0.395	0.77(0.42, 1.42)
Neonatal survival	21(84.0%)	21(84.0%)	> 0.999	1.00(0.79, 1.27)
**Therapeutic cerclage**	**(*n* = 25)**	**(*n* = 23)**		
Gestational latency, week	14.04 ± 2.91	14.29 ± 3.88	0.805	0.25(−1.74, 2.23)
GA at delivery, week	38.29(37.36, 39.08)	38.86(38.14, 39.57)	0.160	0.43(−0.15, 1.28)
GA at delivery < 28 weeks	0(0.0%)	1(4.3%)	0.479	/^b^
GA at delivery < 32 weeks	0(0.0%)	1(4.3%)	0.479	/^b^
GA at delivery < 34 weeks	0(0.0%)	2(8.7%)	0.224	/^b^
GA at delivery < 37 weeks	2(8.0%)	3(13.0%)	0.660	1.63(0.30, 8.90)
PPROM	0(0.0%)	2(8.7%)	0.224	/^b^
PROM	5(20.0%)	6(26.1%)	0.616	1.30(0.46, 3.70)
Positive rate of cervical secretion culture after cerclage	1(4.0%)	4(17.4%)	0.180	4.35(0.52, 36.11)
Post-cerclage CCL, cm	3.03 ± 0.61	2.89 ± 0.52	0.409	−0.14(−0.47, 0.19)
Post-cerclage CCL ≥ 2 cm	24(96.0%)	22(95.7%)	> 0.999	1.00(0.89, 1.12)
Chorioamnionitis	2(8.0%)	5(21.7%)	0.237	2.72(0.58, 12.66)
Neonatal survival	25(100.0%)	22(95.7%)	0.479	0.96(0.88, 1.04)

*GA* gestational age, *PPROM* preterm premature rupture of membranes, *PROM* premature rupture of membranes, *CCL* cervical closure length, *RR* relative risk, *CI* confidence interval

^a^Values are given as Mean ± SD, Median (Q1, Q3), or as number (percentage)

^b^RR not calculable

The rates of PROM and PPROM were reported in Table 2 and did not differ significantly between groups. No other serious maternal adverse events were reported.

A supporting ITT analysis, in which two participants were censored due to early study discontinuation, is presented in Supplementary Fig. 1.

A supporting ITT analysis is presented in Supplementary Fig. 2.

**Fig. 3:**
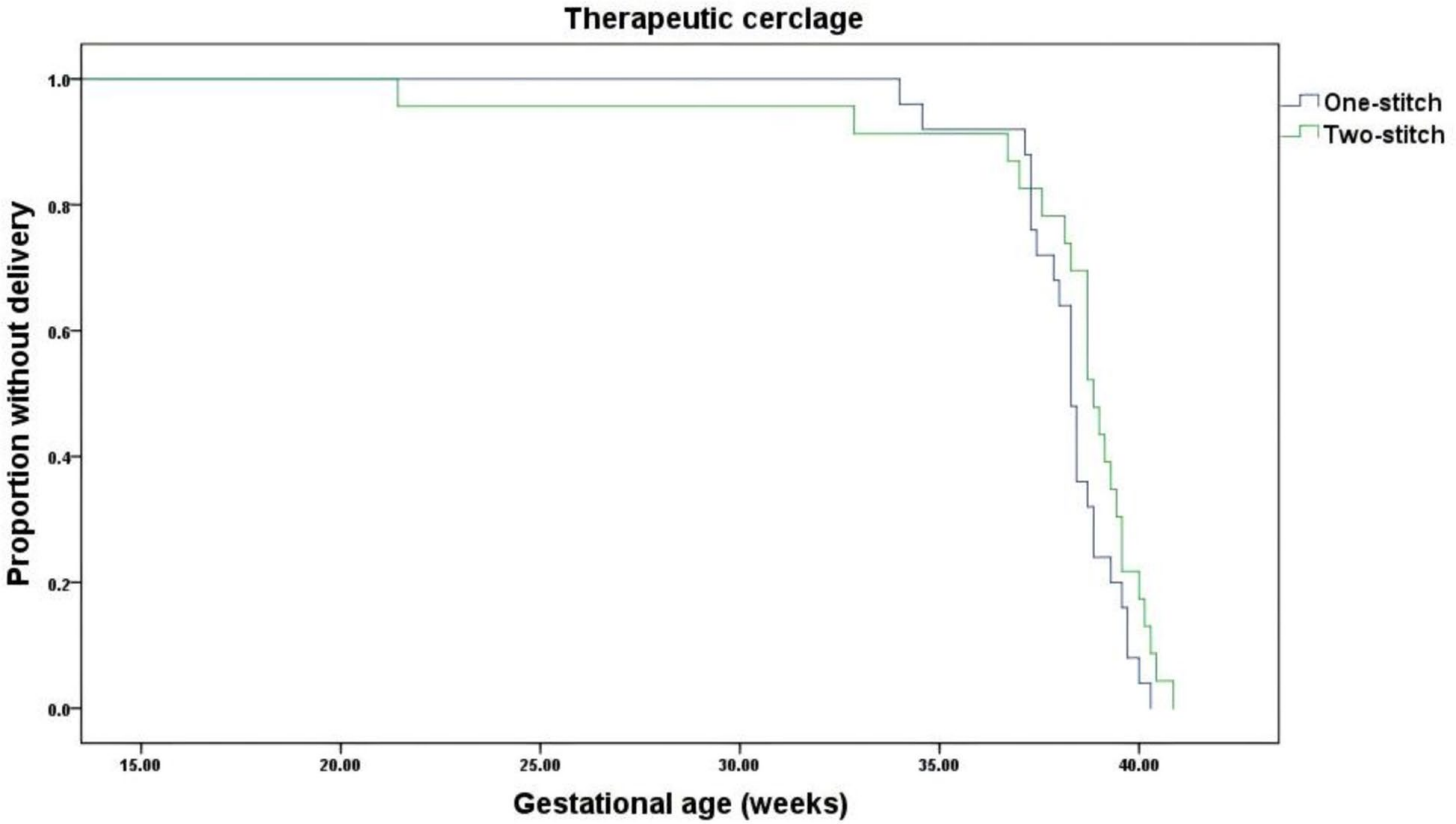
Kaplan–Meier curves of GA at delivery for the therapeutic trial (PP Analysis)

**Fig. 4:**
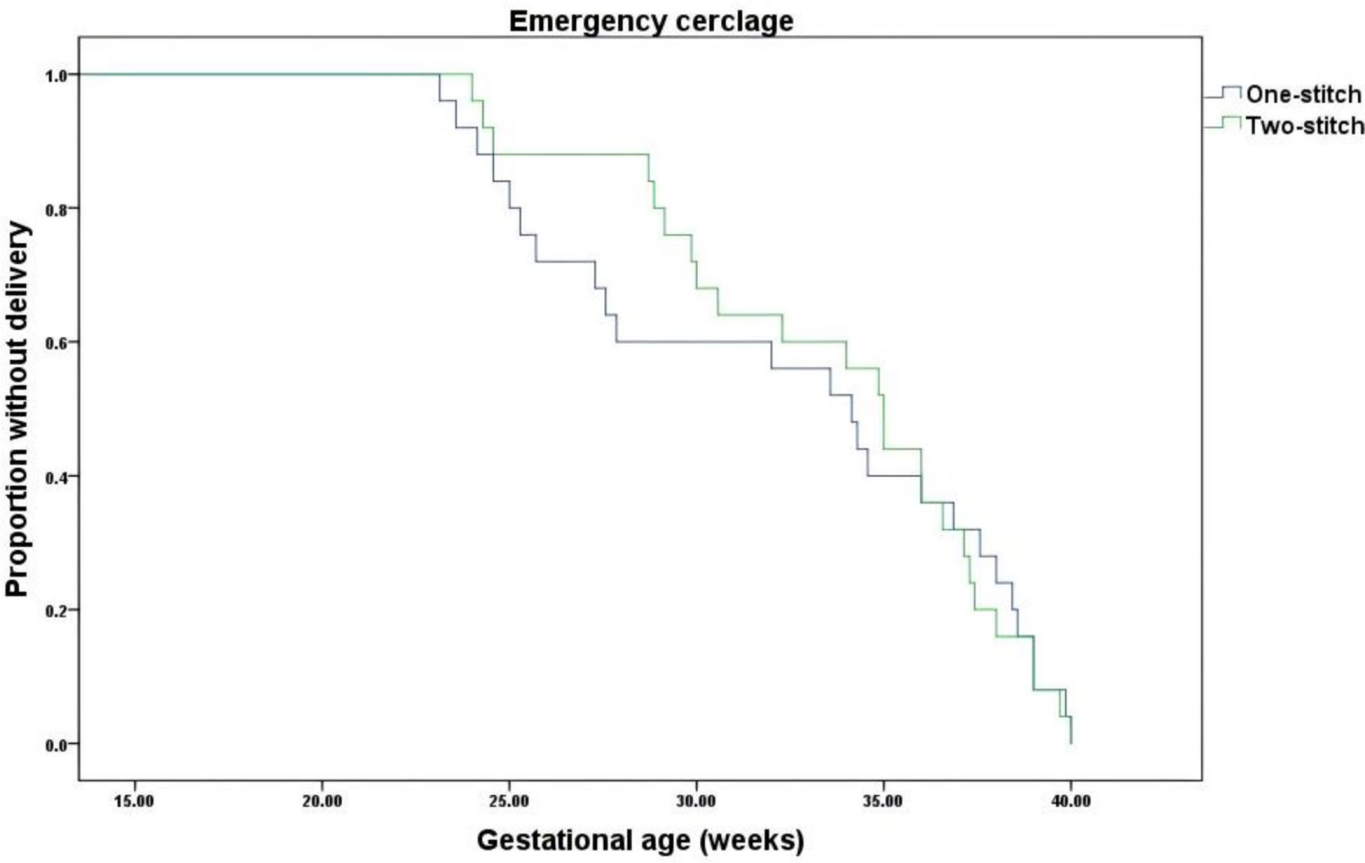
Kaplan–Meier curves of GA at delivery for the emergency trial (PP Analysis)

The Kaplan–Meier survival analysis of the PP population demonstrated no statistically significant difference in the proportion remaining undelivered between the two-stitch and one-stitch groups undergoing therapeutic cervical cerclage (Log-rank χ^2^ = 2.683, df = 1, *P* = 0.101) (Fig. 3). Similarly, for emergency cerclage procedures, analysis of the PP population showed that the two-stitch technique did not result in a significantly higher probability of remaining undelivered compared to the one-stitch technique (Log-rank χ^2^ = 0.022, df = 1, *P* = 0.881) (Fig. 4).

A supporting ITT analysis is presented in Supplementary Fig. 3.

**Fig. 5:**
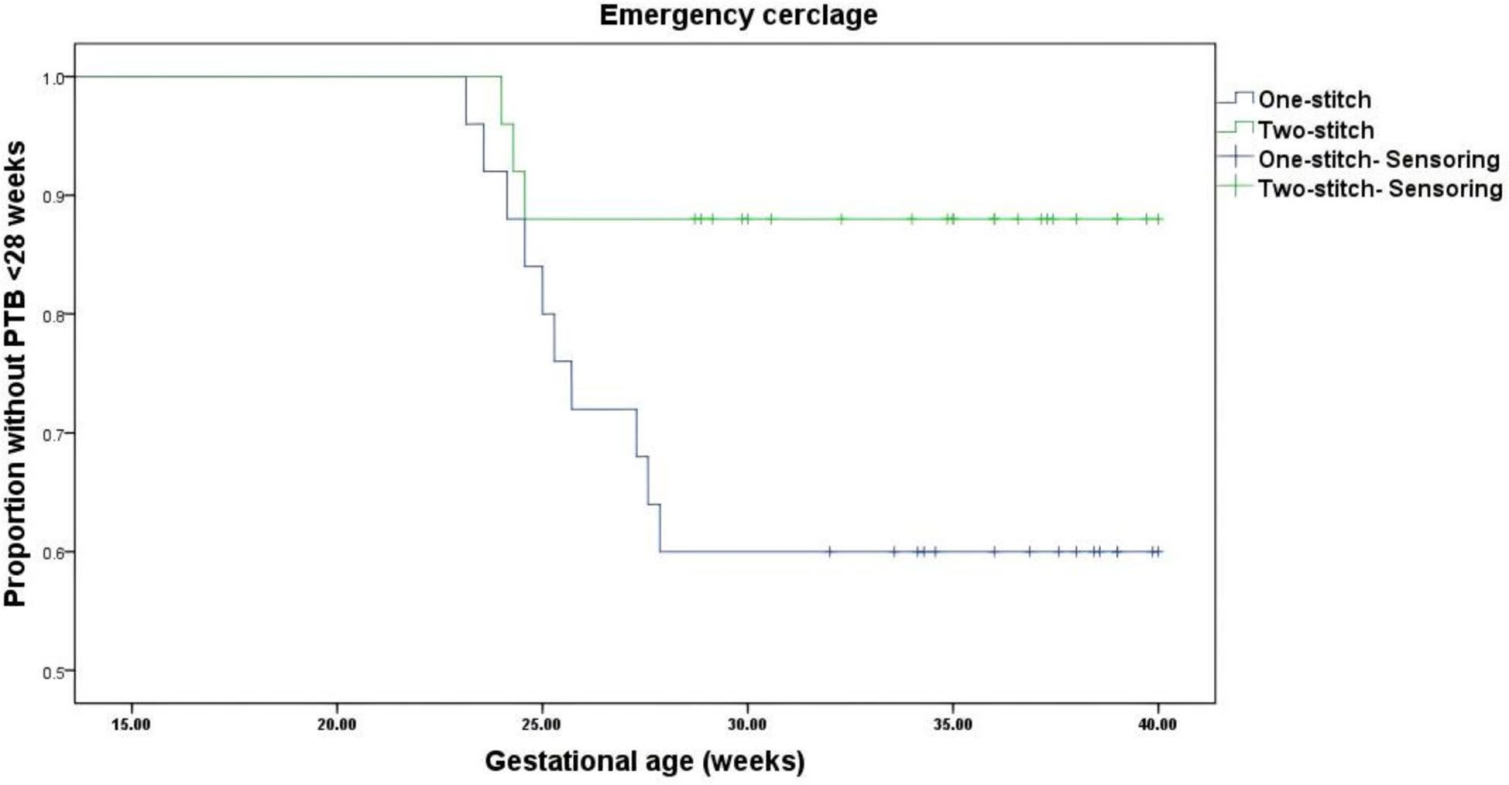
Kaplan–Meier curves of PTB < 28 weeks for the emergency trial (Exploratory PP Analysis)

Notably, an exploratory analysis of the PP population demonstrated a significantly lower proportion of women developing PTB < 28 weeks after emergency cervical cerclage in the two-stitch group versus the one-stitch group (Log-rank χ^2^ = 4.602, df = 1, *P* = 0.032) (Fig. 5).

In the PP analysis, no statistically significant differences were observed between the two-stitch and one-stitch cervical cerclage groups in either the therapeutic or emergency subgroups regarding neonatal outcomes of live births, including live birth weight, Apgar scores < 8 score, NICU admission rate, NICU admission days, or the incidence of necrotizing enterocolitis, respiratory distress syndrome, intraventricular hemorrhage, retinopathy of prematurity, bronchopulmonary dysplasia, and neonatal sepsis (all P > 0.05; see Table 3).

**Table 3:** Neonatal outcomes of live births by suture technique^a^

Variable	One-stitch	Two-stitch	P value	RR or median difference or mean difference (95% CI)
**Emergency cerclage**	**(*n* = 21)**	**(*n* = 21)**		
Birthweight of live births,g	2254.05 ± 984.16	2370.24 ± 774.13	0.673	116.19(−436.04, 668.43)
Very low birth weight (1000-1499 g)	3(14.3%)	4(19.0%)	> 0.999	1.33(0.34, 5.24)
Extremely low birth weight (< 1000 g)	3(14.3%)	0(0.0%)	0.232	/^b^
Apgar score (1 min) < 8 score	3(14.3%)	0(0.0%)	0.232	/^b^
Apgar score (5 min) < 8 score	0(0.0%)	0(0.0%)	/	/^b^
Apgar score (10 min) < 8 score	0(0.0%)	0(0.0%)	/	/^b^
NICU admission	9(42.9%)	6(28.6%)	0.334	0.67(0.29, 1.54)
NICU admission days, d	45.33 ± 37.68	36.00 ± 26.94	0.611	−9.33(−47.99, 29.32)
Necrotizing Enterocolitis	4(19.0%)	1(4.8%)	0.343	0.25(0.03, 2.05)
Respiratory Distress Syndrome	7(33.3%)	4(19.0%)	0.292	0.57(0.20, 1.67)
Intraventricular hemorrhage	7(33.3%)	3(14.3%)	0.147	0.43(0.13, 1.44)
Retinopathy of Prematurity	3(14.3%)	3(14.3%)	> 0.999	1.00(0.23, 4.40)
Bronchopulmonary disease	4(19.0%)	0(0.0%)	0.107	/^b^
Neonatal sepsis	1(4.8%)	1(4.8%)	> 0.999	1.00(0.07, 14.95)
**Therapeutic cerclage**	**(*n* = 25)**	**(*n* = 22)**		
Birthweight of live births,g	3139.40 ± 492.53	3191.14 ± 470.72	0.715	51.74(−232.33, 335.81)
Very low birth weight (1000-1499 g)	0(0.0%)	0(0.0%)	/	/^b^
Extremely low birth weight (< 1000 g)	0(0.0%)	0(0.0%)	/	/^b^
Apgar score (1 min) < 8 score	1(4.0%)	0(0.0%)	> 0.999	/^b^
Apgar score (5 min) < 8 score	0(0.0%)	0(0.0%)	/	/^b^
Apgar score (10 min) < 8 score	0(0.0%)	0(0.0%)	/	/^b^
NICU admission	3(12.0%)	0(0.0%)	0.237	/^b^
NICU admission days, d	5.67 ± 0.58	0	/	/^b^
Necrotizing Enterocolitis	0(0.0%)	0(0.0%)	/	/^b^
Respiratory Distress Syndrome	0(0.0%)	0(0.0%)	/	/^b^
Intraventricular hemorrhage	2(8.0%)	1(4.5%)	> 0.999	0.57(0.06, 5.85)
Retinopathy of Prematurity	0(0.0%)	0(0.0%)	/	/^b^
Bronchopulmonary disease	0(0.0%)	0(0.0%)	/	/^b^
Neonatal sepsis	0(0.0%)	1(4.5%)	0.468	/^b^

*NICU* neonatal intensive care unit, *RR* relative risk, *CI* confidence interval

^a^Values are given as Mean ± SD, Median (Q1, Q3), or as number (percentage)

^b^RR or median difference not calculable

## Discussion

The evidence for the two-stitch cerclage remains inconclusive [5, 7–10]. The cerclage indication is a potential critical effect modifier, yet this hypothesis lacks validation in prospective studies specifically designed with indication-based stratification.

This stratified, exploratory pilot randomized controlled trial provides the prospective evidence comparing two-stitch and one-stitch cerclage. Our primary finding is that the two-stitch technique did not demonstrate superiority over the one-stitch technique for preventing spontaneous PTB < 34 weeks, regardless of whether the indication was therapeutic or emergency. However, our study revealed a nuanced, indication-dependent pattern. In the emergency cerclage cohort, a pre-specified exploratory analysis revealed a notable signal: the two-stitch technique was associated with a 70% reduction in the incidence of extreme PTB < 28 weeks (RR 0.30, 95% CI 0.09–0.96, p = 0.024). This association was consistent across both PP and conservative ITT analyses. It is critical to state that this is a secondary, exploratory finding from an underpowered study, and the nominally significant p-value should be interpreted with caution; nonetheless, the potential clinical impact of reducing extreme prematurity makes this a hypothesis worthy of serious consideration and future validation. Some scholars refer to the distance from the cerclage suture to the external cervical os as the "cerclage height" [14]. This "cerclage height" has been shown to positively correlate with the prolongation of gestation achieved after cervical cerclage [14, 15]. Research indicates that a "cerclage height" exceeding 2 cm is associated with a greater reduction in PTB risk compared to a smaller height [15]. Our findings demonstrate that the two-stitch cerclage was associated with a significantly higher proportion of patients achieving a CCL ≥ 2 cm following emergency cerclage (84.0% vs 56.0%; RR 1.50, 95% CI 1.02–2.21; *P* = 0.031). We speculate that this superior anatomical restoration of the cervical canal might have contributed to the observed risk reduction for PTB < 28 weeks by potentially enhancing mechanical support and facilitating the reformation of a functional mucus plug. However, this proposed pathway remains speculative and requires direct validation in future studies designed to assess such mechanistic outcomes.

Conversely, within the therapeutic cohort, findings from this trial align with the primary outcome and prior literature [5, 8, 9], suggesting no added benefit of a second suture when the cervix is shortened but not dilated. The consistent lack of difference across outcomes, including anatomical restoration (CCL ≥ 2 cm), generates the hypothesis that the two-stitch technique may be superfluous in this clinical scenario, a notion that future larger trials could confirm.

To rationalize these indication-dependent findings, we propose a speculative two-phase biomechanical model. We emphasize that this model is hypothetical and not directly validated by the current data; it is intended purely as a conceptual framework to guide future research.

### Phase I (Functional Compensation)

This phase corresponds to the ultrasound-indicated group. It is theorized to be characterized by cervical extracellular matrix (ECM) degradation and remodeling mediated by the MAPKs-MMPs/TIMPs signaling cascade [16–18]. This process is thought to lead to tissue dissociation and cervical softening, yet the collagen fiber network is presumed to retain sufficient integrity and continuity to maintain baseline circumferential tension. We speculate that during this stage, a one-stitch cerclage might be sufficient to achieve the therapeutic threshold, avoiding the potential infection risk associated with an additional suture placement.

### Phase II (Structural Decompensation)

**Fig. 6:**
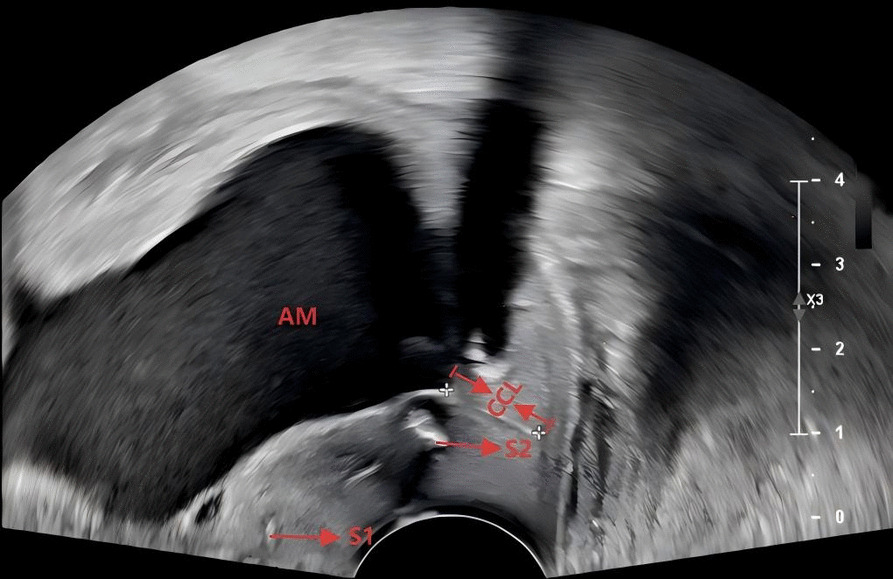
Schematic diagram illustrating the hypothetical reinforcing mechanism of a two-stitch cerclage. When the first suture (S1) fails, the second suture (S2) maintains cervical closure length (CCL) and prevents descent of the amniotic membrane (AM). Abbreviations: S1, first suture; S2, second suture; AM, amniotic membrane; CCL, cervical closure length

This phase corresponds to the physical examination-indicated cohort. We postulate that it involves progression from microstructural decoupling to macroscopic failure. Dilation of the external cervical os marks an irreversible biomechanical threshold. Membrane exposure exacerbates the imbalance within the TIMPs/MMPs system, accelerating cervical ECM degradation [19–21], thereby increasing cervical distensibility and ultimately leading to loss of circumferential tension. Within this conceptual framework, we hypothesize that the two-stitch technique might generate synergistic hoop stresses that could help reconstitute the anatomical and mechanical integrity of the cervix [22]. This potential mechanism might explain the observed reduction in extreme PTB risk observed with two-stitch cerclage in the emergency group. Furthermore, previous studies report cases requiring repeat cerclage after one-stitch procedures due to membranes descending below the suture [23]; in our clinical experience with the two-stitch technique, we noted that when membranes prolapse below the first suture, the second suture appeared to maintain mechanical support, closing the external os and preventing membrane exposure (Fig. 6). This model, while speculative, provides a potential biomechanical rationale for why an additional suture might confer benefit only when the cervical architecture has undergone significant failure.

**Fig. 7:**
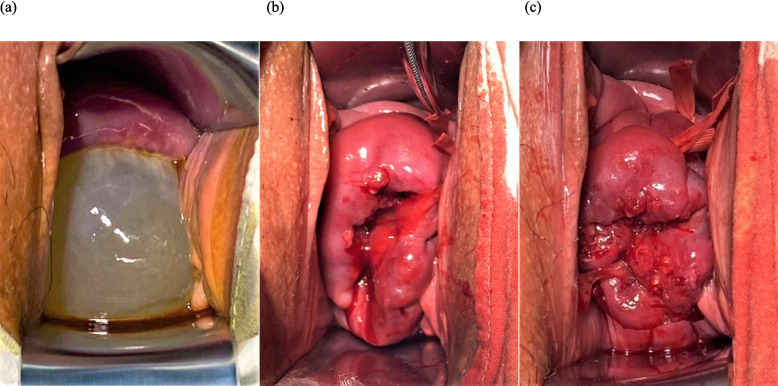
Schematic diagram illustrating cervical changes during the two-stitch cerclage procedure. **a** Dilated cervix with bulging membrane before cerclage. **b** The external os of the cervix was still dilated in the shape of a horn mouth even after placing the first suture at a sufficiently high level. **c** After placing a second suture close to the external os, the anatomical structure of the cervical canal was better restored

Infection is a significant factor in PTB [24]. In cervical insufficiency, the risk of intra-amniotic infection rises significantly when cervical dilation > 2 cm [11]. The cervical mucous plug serves as a critical barrier, protecting the amniotic cavity against ascending infection [25]. However, in cases of cervical dilation or membrane prolapse (indicating loss of the mucous plug barrier), a suture placed solely at the level of the internal os may be insufficient to establish an effective barrier against ascending pathogens [26] —as shown in Fig. 7a depicting a patient with cervical dilation, where even after placing the first suture close enough to the internal os, the thin tissue of the external os remained dilated in a horn-shaped configuration (Fig. 7b). This limitation may potentially compromise surgical outcomes. This study observed that the two-stitch technique achieves superior anatomical restoration of the cervical canal (CCL ≥ 2 cm). We therefore hypothesize that performing two-stitch cerclage under these circumstances may be beneficial: by placing a second suture to achieve superior anatomical restoration of the cervical canal (Fig. 7c) [10], this technique not only provides enhanced anatomical reconstruction but also promotes reformation of the mucous plug. However, this hypothesis requires validation through future studies specifically designed to directly measure markers of intra-amniotic infection or evaluate characteristics of the mucous plug.

When interpreting our findings in the context of contemporary practice, it is important to acknowledge that cervical pessary represents a non-surgical alternative for managing a short cervix [27]. A recent study on a novel pessary insertion technique showed superior efficacy in preventing preterm birth compared to the standard approach [28]. While the cervical pessary is an established non-surgical option for women with a short cervix and closed os, its role in the context of cervical dilation remains investigational. In cases of cervical dilation with or without membrane prolapse—as enrolled in our emergency cerclage cohort—the primary pathophysiological challenge is the loss of structural integrity, which necessitates direct mechanical reinforcement typically provided by cerclage. Therefore, our trial focused on women for whom surgical cerclage was the clinically indicated intervention. Future studies could explore whether a subset of women with minimal dilation might benefit from pessary, but such an approach would require careful validation against the established efficacy of emergency cerclage. Similarly, the evolving evidence regarding adjunctive therapies, such as antenatal corticosteroids, underscores the complexity of managing PTB. While our institutional protocol ensured balanced use between groups, recent debates highlight that the benefits of antenatal corticosteroids may vary in specific subpopulations [29–31]. This reinforces the need for standardized, refined protocols in future trials to precisely delineate the independent contribution of the surgical technique itself.

The interpretation of our findings must be considered in the context of several critical limitations, many of which align with the inherent challenges of an exploratory surgical trial. First, this was a pilot study with a sample size calculated based on an optimistic effect size from a non-randomized study. Consequently, it was inherently underpowered for its primary endpoint, and the results, particularly the nominally significant secondary outcomes, must be interpreted as strictly hypothesis-generating. Second, a key limitation is the risk of bias due to the inability to blind the surgical team and the potential for unblinding of other personnel—a challenge inherent to surgical trials. Although procedures were implemented to maintain blinding of outcome assessors, we cannot guarantee perfect maintenance throughout the study. It should be noted that both the primary outcome and key secondary outcomes were determined using objective criteria. This objectivity helps mitigate the potential for detection bias in these critical endpoints. Third, the single-center design and performance of all procedures by one surgeon, while maximizing technical consistency, markedly limits the generalizability of our findings. Furthermore, the absence of adjustments for multiple comparisons increases the risk that the observed association with PTB < 28 weeks represents a type I error. Finally, the absence of pre-specified subgroup analyses limits the interpretability of our findings and precludes the identification of patient subgroups that might derive the greatest benefit from the two-stitch technique.

Despite these limitations, this trial provides crucial insights for future research. The stratified randomization by indication proved feasible for addressing distinct clinical questions. Moreover, the standardized surgical technique, as implemented here, offers a template for procedural consistency essential for future multicenter trials, which must be adequately powered and include pre-specified, protected analyses to confirm or refute the signals generated by this exploratory study.

## Conclusions

In conclusion, this pilot trial does not support the routine use of the two-stitch cerclage technique over the one-stitch technique for preventing PTB < 34 weeks. However, in the setting of emergency cerclage, it generates the clinically important hypothesis that the two-stitch approach may be more effective in reducing the risk of extreme prematurity before 28 weeks. This potential benefit, coupled with a comparable safety profile, should not dictate current practice but must be rigorously evaluated in a large, multicenter, definitive RCT, adequately powered to confirm or refute this signal and to identify the patient subgroups most likely to benefit.

## Supplementary Information

Supplementary Material 1.

Supplementary Material 2.

Supplementary Material 3.

Supplementary Material 4.

Supplementary Material 5.

Supplementary Material 6.

## Data Availability

The datasets used and/or analyzed during the current study are available from the corresponding author on reasonable request.

## Abbreviations

BMI: Body Mass Index
CCL: Cervical Closure Length
CI: Confidence Interval
CRL: Crown-Rump Length
CRP: C-Reactive Protein
ECM: Extracellular Matrix
GA: Gestational Age
ITT: Intention-to-treat
NICU: Neonatal Intensive Care Unit
PP: Per-protocol
PPROM: Preterm Premature Rupture of Membranes
PROM: Premature Rupture of Membranes
PTB: Preterm Birth
RCT: Randomized Controlled Trial
RR: Relative Risk
SD: Standard Deviation
