# Supplementary material for: Two-stitch versus one-stitch cervical cerclage in women with high risk for preterm birth: a stratified exploratory randomized controlled trial in China
Source: BMC Pregnancy Childbirth. 2026 Feb 16;26:316. doi: 10.1186/s12884-026-08809-8 (PMC13014719; doi:10.1186/s12884-026-08809-8)
Supplement: Supplementary file 1 — Supplementary Material 1. [file 12884_2026_8809_MOESM1_ESM.doc]

**Protocol Title:** Two-stitch versus one-stitch cervical cerclage in women with cervical insufficiency: a stratified randomised controlled trial in China

**Protocol Version:** 2.0
**Date:** April 10, 2022
**Trial Registration:** Chinese Clinical Trial Registry (ChiCTR2200058540)

**1.0 Background and Rationale**

Preterm birth (PTB) remains the leading cause of neonatal mortality worldwide. Cervical insufficiency is a significant aetiology, and transvaginal cervical cerclage is the standard surgical treatment. Despite widespread use, a substantial proportion of women, particularly those requiring emergency cerclage for cervical dilatation, still deliver preterm. The two-stitch cerclage technique has been proposed as a strategy to provide enhanced mechanical support and potentially restore the cervical mucus plug barrier, thereby reducing the risk of ascending infection and PTB. However, current evidence is derived from limited, heterogeneous retrospective studies with conflicting conclusions. No prior randomised controlled trials have stratified participants by surgical indication to evaluate the efficacy of this technique. This trial aims to provide high-quality evidence on whether a two-stitch cerclage is superior to a traditional one-stitch technique in prolonging pregnancy and improving neonatal outcomes, specifically within therapeutic and emergency cerclage cohorts.

**2.0 Objectives**
2.1 **Primary Objective:** To compare the incidence of spontaneous preterm birth at <34 weeks of gestation between women receiving two-stitch versus one-stitch McDonald cerclage, stratified by indication (therapeutic vs. emergency).
2.2 **Secondary Objectives:** To compare between groups:
Gestational age at delivery.
Spontaneous preterm birth at <28 weeks, <32 weeks and <37 weeks.
Gestational latency (interval from cerclage to delivery).
Postoperative cervical length.
Incidence of preterm premature rupture of membranes (PPROM), chorioamnionitis, and positive cervical culture.
Neonatal survival and morbidity (e.g., NICU admission, RDS, IVH, NEC, sepsis).
Maternal complications.

**3.0 Trial Design**
A single-centre, stratified, parallel-group, randomised, controlled, superiority trial. The study will be conducted at Fujian Maternity and Child Health Hospital, Fuzhou, China.

**4.0 Participants, Inclusion and Exclusion Criteria**
4.1 **Inclusion Criteria (must meet one category):**
**Therapeutic Cerclage Cohort:** Singleton pregnancy at 16-28 weeks gestation with:
(a) History of spontaneous PTB <34 weeks AND current cervical length (CL) ≤25 mm at 16-28 weeks; OR
(b) No prior PTB BUT documented progressive cervical shortening with CL ≤25 mm at 16-28 weeks
**Emergency Cerclage Cohort:** Singleton pregnancy at 16-28 weeks gestation with painless cervical dilatation, with or without membrane prolapse.
4.2 **Exclusion Criteria:**
(a) Transabdominal cerclage;

(b) Using a pessary for cervical shortening;

(c) Fetal anomalies;

(d) Medically indicated preterm delivery;

(e) Lost to follow-up or incomplete documentation of delivery outcomes.

**5.0 Interventions**
5.1 **Preoperative preparation:** The basic condition of the patient was fully assessed before surgery, and preoperative examinations were completed to exclude surgical contraindications. The patient was informed of the condition and the risks related to the surgery, and a surgical informed consent form was signed.

5.2 **Surgical procedure:** All patients underwent the McDonald cervical cerclage procedure performed by the same experienced chief gynecologist and obstetrician, using Johnson & Johnson RS22 suture material. During the surgery, the cervix was fully exposed, and several oval forceps were used simultaneously to pull the cervix outward to retract the cervical os. If the amniotic sac protruded beyond the external cervical os, it was reduced to the level of the internal cervical os. In the one-stitch group, the Johnson & Johnson RS22 suture was used to perform a circular continuous suture and knot at the level of the cervicovaginal junction. In the two-stitch group, the first cerclage suture was performed in the same manner as the one-stitch group, and the second suture was performed again in a circular suture near the external cervical os below the first suture to close the external cervical os.

5.3 **Postoperative management:** Patients were placed in a Trendelenburg position for bed rest after surgery. Perioperative antibiotics were used to prevent infection, and external genital hygiene was maintained. Infection indicators, uterine contractions, vaginal discharge, and subjective symptoms of the patient were closely monitored. The changes in the cervix were dynamically monitored by ultrasound. Appropriate tocolytic agents were used according to gestational age and uterine contraction symptoms. The cervical cerclage was usually removed at 36-37 weeks of gestation. In case of definite signs of infection, preterm premature rupture of membranes (PPROM), inevitable miscarriage or preterm labor unresponsive to tocolytic agents, the cerclage should be urgently removed in the emergency department. The timing of cerclage removal should be grasped to reduce the occurrence of cervical laceration.

**6.0 Outcomes**
6.1 **Primary Outcome:** Spontaneous preterm birth at <34^+0^ weeks gestation.
6.2 **Secondary Outcomes:** As listed in section 2.2. Gestational dating was standardized using first-trimester crown-rump length (CRL) measurements (11-13 weeks). When discrepancy >7 days existed between last menstrual period (LMP) and ultrasound dating, CRL-based dating was prioritized.

**7.0 Sample Size Calculation**

Based on historical data[1], the expected incidence of PTB <34 weeks was 89.5% in the emergency one-stitch group and 42.1% in the emergency two-stitch group. Assuming a power of 90% and a two-sided alpha of 0.05, a sample size of 44 patients was required for the emergency stratum. Accounting for a 5% dropout rate, a minimum of 46 patients per stratum (therapeutic and emergency) will be recruited, leading to a total target enrolment of 100 participants (50 per stratum, 25 per treatment arm within each stratum).

**8.0 Randomisation and Blinding**
**8.1 Randomisation**

Participants were primarily stratified by clinical indication (Therapeutic cerclage cohort vs. Emergency cerclage cohort, n=50 per stratum). Within each stratum, they were randomly assigned (1:1) to receive either the one-stitch or two-stitch technique (n=25 per group) using a computer-generated random number sequence (created with SPSS 26.0) with permuted blocks of sizes 4 and 6.

Allocation concealment was implemented using sequentially numbered, opaque, sealed envelopes. For each eligible participant, an independent research nurse provided the corresponding envelope directly in the operating room. The envelope was opened by the surgeon only after anaesthesia preparation and the final decision to proceed with surgery. The surgeon had no access to the master allocation sequence, and only one envelope was present in the operating room at any time. Stratum-specific randomisation lists were maintained independently to prevent cross-stratum contamination.

**8.2 Blinding**

Due to the procedural nature of the intervention, the operating surgeons could not be blinded to the group assignment. However, the following blinding procedures were implemented:

Participants and Postoperative Nursing Staff: Were blinded to group assignment. This was achieved through standardised postoperative care protocols and by omitting documentation of the number of sutures in the patient's accessible medical records.

Outcome Assessors: Were blinded to the treatment allocation. The primary outcome (spontaneous preterm birth at <34 weeks) was determined based on objective criteria not subject to interpretation based on the surgical technique.

Furthermore, the researcher who managed the randomisation information did not participate in any subsequent trial stages. Although unblinded, surgeons were mandated to follow standardised operative checklists and were excluded from outcome evaluations.

This design ensured that potential confounding from clinical indication type was controlled through stratification, while preserving the properties of random allocation within each prognostic subgroup.

**9.0 Data Collection and Management**

Data will be collected prospectively using standardised case report forms (CRFs) and entered into a secure electronic database. All patient identifiers will be removed. Data quality will be ensured through regular audits and validation checks.

**10.0 Statistical Analysis Plan**

**10.1 Analysis Principles**

The primary analysis will be performed according to the intention-to-treat (ITT) principle, including all randomly assigned participants in the groups to which they were originally allocated.

A supporting per-protocol (PP) analysis will also be conducted, which excludes participants who did not receive the allocated intervention or who had major protocol violations.

A two-sided p-value of less than 0.05 will be considered statistically significant for the primary outcome. All analyses will be performed using SPSS software (version 26.0, IBM SPSS, Inc, Armonk, NY).

**10.2 Handling of Missing Data**

For the primary and secondary outcome, if data are missing, a conservative approach will be applied by imputing the missing participants as having experienced the event in the primary ITT analysis.

The PP analysis will simply exclude participants with missing primary outcome data.

**10.3 Analysis of Baseline Characteristics**

Baseline maternal demographics and clinical characteristics will be summarized by treatment group within each stratum (Therapeutic and Emergency).

Continuous variables will be assessed for normality using the Shapiro-Wilk test.

Normally distributed data will be presented as mean ± standard deviation (SD) and compared between groups using Independent Samples t-tests.

Non-normally distributed data will be expressed as median (Q1, Q3) and compared using the Wilcoxon rank-sum test (Mann-Whitney U test).

Categorical variables will be presented as number (percentage) and compared using:

Chi-square (χ²) test when all expected frequencies are ≥5.

Fisher's exact test when any expected frequency is <5.

**10.4 Analysis of Outcomes**

10.4.1 Primary Outcome

The primary outcome, spontaneous PTB <34 weeks, is a dichotomous variable.

It will be compared between the two-stitch and one-stitch groups within each stratum using a Chi-square test or Fisher's exact test as appropriate.

The relative risk (RR) with a 95% confidence interval (CI) will be calculated as the primary measure of effect size.

10.4.2 Secondary Outcomes

Dichotomous outcomes (e.g., PTB at <28, <32, <37 weeks; PPROM; chorioamnionitis; neonatal survival; etc.) will be analyzed similarly to the primary outcome, presenting RR with 95% CI and using Chi-square or Fisher's exact test.

Continuous outcomes:

Normally distributed variables (e.g., gestational age at delivery in the emergency cohort PP analysis) will be presented as mean ± SD and analyzed with Independent Samples t-tests, reporting the mean difference with 95% CI.

Non-normally distributed variables (e.g., gestational latency, GA at delivery in the therapeutic cohort) will be presented as median (Q1, Q3) and analyzed with the Wilcoxon rank-sum test, with the median difference and its 95% CI calculated via the Hodges-Lehmann method.

**10.5 Time-to-Event Analysis**

Time-to-event data (gestational age at delivery) were analyzed using Kaplan-Meier survival curves and compared with the log-rank test. Participants who did not experience the primary event (spontaneous preterm birth <34 weeks) were administratively censored. Specifically, censoring occurred at the gestational age of term delivery, medically indicated (iatrogenic) delivery, study withdrawal (e.g., due to protocol violations or subsequent ineligibility), or loss to follow-up (last known gestational age with confirmed pregnancy status).

**10.6 Subgroup and Other Analyses**

The primary analysis is stratified by indication (Therapeutic vs. Emergency), which serves as a pre-specified subgroup analysis.

No other subgroup or sensitivity analyses were pre-specified in the statistical analysis plan for this initial trial.

No interim analyses for efficacy or futility were planned or conducted.

**10.7 Data Presentation**

All results will be presented with the exact p-values.

Effect estimates (RR, mean difference, median difference) will be reported with their 95% CIs.

The analysis will be reported in accordance with the CONSORT guidelines.

**11.0 Ethics and Monitoring**

The study protocol was approved by the Institutional Ethics Committee of Fujian Maternity and Child Health Hospital (Approval No: 2022YJ012). Written informed consent will be obtained from all participants prior to any study procedures. The trial will be monitored by an independent data and safety committee to ensure participant safety and data integrity.

**12.0 Protocol Amendments**

Any amendments to the protocol requiring changes to study design, procedures, or informed consent will be submitted to the Ethics Committee for approval prior to implementation. Changes will also be updated in the clinical trial registry.

**13.0 Consent and Confidentiality**

Participant confidentiality will be strictly maintained. All data will be de-identified and stored securely. Consent forms will be stored separately from the research data.

1. Xu ZM, Zhang J, Hong XL, Liu J, Yang ZZ, Pan M**: Comparison of two stitches versus one stitch for emergency cervical cerclage to prevent preterm birth in singleton pregnanci**es*. International journal of gynaecology and obstetrics: the official organ of the International Federation of Gynaecology and Obstetric*s 2023**, 1**60(1):98-105.
