# Supplementary material for: Two-stitch versus one-stitch cervical cerclage in women with high risk for preterm birth: a stratified exploratory randomized controlled trial in China
Source: BMC Pregnancy Childbirth. 2026 Feb 16;26:316. doi: 10.1186/s12884-026-08809-8 (PMC13014719; doi:10.1186/s12884-026-08809-8)
Supplement: Supplementary file 5 — Supplementary Material 5. [file 12884_2026_8809_MOESM5_ESM.doc]

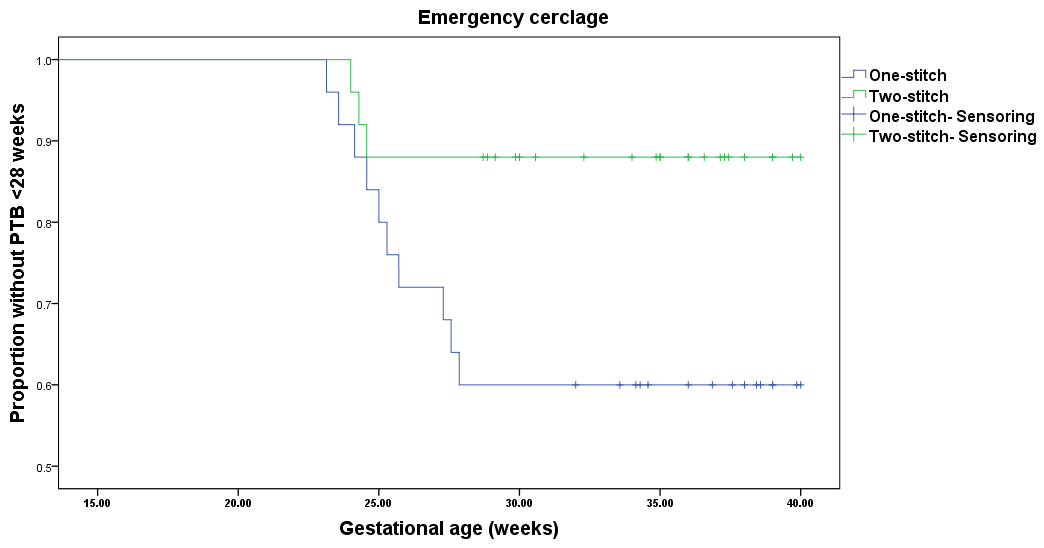


**Supplementary Figure 3 Kaplan–Meier curves of PTB<28 weeks for the emergency trial (Log-rank χ²=4.602, df=1, P=0.032) (Exploratory ITT Analysis).**
