# Supplementary material for: Two-stitch versus one-stitch cervical cerclage in women with high risk for preterm birth: a stratified exploratory randomized controlled trial in China
Source: BMC Pregnancy Childbirth. 2026 Feb 16;26:316. doi: 10.1186/s12884-026-08809-8 (PMC13014719; doi:10.1186/s12884-026-08809-8)
Supplement: Supplementary file 6 — Supplementary Material 6. [file 12884_2026_8809_MOESM6_ESM.doc]

**Supplementary Table 1. Comparisons of Pregnancy and Neonatal Outcomes Between Suture Techniques: Intention-to-Treat (ITT) and Per-Protocol (PP) Analyses in the Therapeutic and Emergency Cerclage Cohorts**

**Supplementary Table 1a. Emergency Cerclage Cohort: Outcomes by Suture Technique (ITT and PP Populations)**

| **Outcome Measure** | **One-stitch**  **(ITT) n=25** | **Two-stitch**  **(ITT) n=25** | **P value**  **(ITT)** | **Effect Size (95% CI) (ITT)** | **One-stitch**  **(PP) n=25** | **Two-stitch**  **(PP) n=25** | **P value**  **(PP)** | **Effect Size (95% CI) (PP)** |
| --- | --- | --- | --- | --- | --- | --- | --- | --- |
| ****Primary Outcome**** |  |  |  |  |  |  |  |  |
| GA at delivery <34 weeks | 12 (48.0%) | 10 (40.0%) | 0.569 | RR 0.83 (0.44, 1.57) | 12 (48.0%) | 10 (40.0%) | 0.569 | RR 0.83 (0.44, 1.57) |
| ****Secondary Outcomes**** |  |  |  |  |  |  |  |  |
| GA at delivery <28 weeks | 10 (40.0%) | 3 (12.0%) | 0.024 | RR 0.30 (0.09, 0.96) | 10 (40.0%) | 3 (12.0%) | 0.024 | RR 0.30 (0.09, 0.96) |
| GA at delivery <32 weeks | 10 (40.0%) | 9 (36.0%) | 0.771 | RR 0.90 (0.44, 1.83) | 10 (40.0%) | 9 (36.0%) | 0.771 | RR 0.90 (0.44, 1.83) |
| GA at delivery <37 weeks | 17 (68.0%) | 17 (68.0%) | >0.999 | RR 1.00 (0.68, 1.46) | 17 (68.0%) | 17 (68.0%) | >0.999 | RR 1.00 (0.68, 1.46) |
| PPROM | 7 (28.0%) | 10 (40.0%) | 0.370 | RR 1.43 (0.65, 3.15) | 7 (28.0%) | 10 (40.0%) | 0.370 | RR 1.43 (0.65, 3.15) |
| PROM | 8 (32.0%) | 11 (44.0%) | 0.382 | RR 1.38 (0.67, 2.83) | 8 (32.0%) | 11 (44.0%) | 0.382 | RR 1.38 (0.67, 2.83) |
| Positive rate of cervical secretion culture after cerclage | 8 (32.0%) | 9 (36.0%) | 0.765 | RR 1.13 (0.52, 2.44) | 8 (32.0%) | 9 (36.0%) | 0.765 | RR 1.13 (0.52, 2.44) |
| Post-cerclage CCL ≥2cm | 14(56.0%) | 21(84.0%) | 0.031 | RR 1.50 (1.02, 2.21) | 14(56.0%) | 21(84.0%) | 0.031 | RR 1.50 (1.02, 2.21) |
| Chorioamnionitis | 13 (52.0%) | 10 (40.0%) | 0.395 | RR 0.77 (0.42, 1.42) | 13 (52.0%) | 10 (40.0%) | 0.395 | RR 0.77 (0.42, 1.42) |
| Neonatal survival | 21(84.0%) | 21(84.0%) | >0.999 | RR 1.00(0.79, 1.27) | 21(84.0%) | 21(84.0%) | >0.999 | RR 1.00(0.79, 1.27) |

Supplementary Table 1b. Therapeutic Cerclage Cohort: Outcomes by Suture Technique (ITT and PP Populations)

| **Outcome Measure** | **One-stitch**  **(ITT) n=25** | **Two-stitch**  **(ITT) n=25** | **P value**  **(ITT)** | **Effect Size (95% CI)**  **(ITT)** | **One-stitch**  **(PP) n=25** | **Two-stitch**  **(PP) n=23** | **P value**  **(PP)** | **Effect Size (95% CI) (PP)** |
| --- | --- | --- | --- | --- | --- | --- | --- | --- |
| ****Primary Outcome**** |  |  |  |  |  |  |  |  |
| GA at delivery <34 weeks | 0 (0.0%) | 4 (16.0%) ^a^ | 0.110 | / ^c^ | 0 (0.0%) | 2 (8.7%) | 0.224 | / ^c^ |
| ****Secondary Outcomes**** |  |  |  |  |  |  |  |  |
| GA at delivery <28 weeks | 0 (0.0%) | 3 (12.0%) ^a^ | 0.235 | / ^c^ | 0 (0.0%) | 1 (4.3%) | 0.479 | / ^c^ |
| GA at delivery <32 weeks | 0 (0.0%) | 3 (12.0%) ^a^ | 0.235 | / ^c^ | 0 (0.0%) | 1 (4.3%) | 0.479 | / ^c^ |
| GA at delivery <37 weeks | 2 (8.0%) | 5 (20.0%) ^a^ | 0.417 | RR 2.50 (0.53, 11.70) | 2 (8.0%) | 3 (13.0%) | 0.660 | RR 1.63 (0.30, 8.90) |
| PPROM | 0 (0.0%) | 4 (16.0%) ^a^ | 0.110 | / ^c^ | 0 (0.0%) | 2 (8.7%) | 0.224 | / ^c^ |
| PROM | 5 (20.0%) | 8 (32.0%) ^a^ | 0.333 | RR 1.60 (0.61, 4.22) | 5(20.0%) | 6 (26.1%) | 0.616 | RR 1.30 (0.46, 3.70) |
| Positive rate of cervical secretion culture after cerclage | 1 (4.0%) | 6 (24.0%) ^a^ | 0.098 | RR 6.00 (0.78, 46.29) | 1 (4.0%) | 4 (17.4%) | 0.180 | RR 4.35 (0.52, 36.11) |
| Post-cerclage CCL ≥2cm | 24(96.0%) | 22(88.0%) ^b^ | 0.609 | RR0.92 (0.78, 1.08) | 24(96.0%) | 22(95.7%) | >0.999 | RR1.00 (0.89, 1.12) |
| Chorioamnionitis | 2 (8.0%) | 7 (28.0%) ^a^ | 0.138 | RR 3.50 (0.80, 15.23) | 2 (8.0%) | 5 (21.7%) | 0.237 | RR 2.72 (0.58, 12.66) |
| Neonatal survival | 25(100.0%) | 22(88.0%) ^b^ | 0.235 | RR0.88 (0.76, 1.02) | 25(100.0%) | 22(95.7%) | 0.479 | RR0.96 (0.88, 1.04) |

Abbreviation: ITT, intention-to-treat; PP, per-protocol; GA, gestational age; PPROM, preterm premature rupture of membranes; PROM, premature rupture of membranes; CCL, cervical closure length; RR, relative risk; CI, confidence interval.

^a^ In the ITT analysis, two participants in the therapeutic two-stitch group with missing primary outcome data were imputed as having experienced the event.

^b^ In the ITT analysis, two participants in the therapeutic two-stitch group with missing primary outcome data were imputed as not having experienced the event.

^c^ RR not calculable
